# Clinical and Demographic Profile of COVID-19 Patients: A Tertiary Level Hospital-Based Study From Northeast India

**DOI:** 10.7759/cureus.18881

**Published:** 2021-10-19

**Authors:** Md Jamil, Prasanta K Bhattacharya, Bhupen Barman, Noor Topno, Himesh Barman, Vijay N Nongpiur, Gwenette War, Yasmeen Hynniewta, Bishwajeet Saikia, Narang Naku

**Affiliations:** 1 Department of General Medicine, North Eastern Indira Gandhi Regional Institute of Health and Medical Sciences, Shillong, IND; 2 Internal Medicine, North Eastern Indira Gandhi Regional Institute of Health and Medical Sciences, Shillong, IND; 3 Department of General Surgery, North Eastern Indira Gandhi Regional Institute of Health and Medical Sciences, Shillong, IND; 4 Paediatrics, North Eastern Indira Gandhi Regional Institute of Health and Medical Sciences, Shillong, IND; 5 Department of TB and Respiratory Diseases, North Eastern Indira Gandhi Regional Institute of Health and Medical Sciences, Shillong, IND; 6 Neurology, North Eastern Indira Gandhi Regional Institute of health and Medical Sciences, Shillong, IND; 7 Department of Anatomy, North Eastern Indira Gandhi Regional Institute of Health and Medical Sciences, Shillong, IND

**Keywords:** northeast india, comorbidity, clinical profile, sars-cov-2, covid-19

## Abstract

Background and objective

The coronavirus disease 2019 (COVID-19) outbreak, which was first detected in Wuhan, China, has turned into a rapidly spreading global healthcare crisis. The clinical and laboratory features of COVID-19 are associated with significant regional variations. In this study, we aimed to describe the clinical and demographic profile of COVID-19 patients from a tertiary care hospital in Northeast India.

Materials and methods

This was a hospital-based cross-sectional study that included all laboratory-confirmed COVID-19 cases admitted to the institution from 1st July to 31st October 2020. The information was collected on a predesigned proforma, which included patients' demographic profiles, clinical presentations, and outcomes as per treatment by trained doctors.

Results

The study included 180 laboratory-confirmed COVID-19 cases. A history of contact with laboratory-confirmed COVID-19-affected individuals was found in 92 (51.1%) patients. The median age of the patients was 37.17 years (range: 18-80 years), and there were 104 (57.78%) males in the cohort. Of the total enrolled patients, 102 (56.67%) were asymptomatic from the time of exposure till their admission. The common presenting complaints were fever (n=55, 70.51%), cough (n=42, 53.85%), and shortness of breath (n=32, 42.02%). The case fatality rate among the admitted cases was 15%. Comorbidities were found in 84 (46.67%) patients with the most common one being diabetes mellitus (n=31, 36.9%) followed by hypertension (n=29, 34.52%). Patients with advanced age (more than 60 years) and coexisting comorbidities were at higher risk of progression of disease and death.

Conclusion

The COVID-19 pandemic is not only a huge burden on healthcare facilities but also a significant cause of disruption in societies globally. The majority of the patients with COVID-19 infection presenting to our hospital were young and asymptomatic. Patients of advanced age with comorbidities were found to have more complications. An analysis of the trends related to COVID-19 in different hospital and institutional settings will help to achieve better preparedness and lead to improved patient care to combat the COVID-19 pandemic in a more efficient manner.

## Introduction

Coronavirus disease 2019 (COVID-19) is a viral pneumonia caused by a novel coronavirus (novel CoV) called severe acute respiratory syndrome coronavirus 2 (SARS-CoV-2). SARS-CoV-2 belongs to the same family of viruses as the causative agents of the other two recent outbreaks of viral pneumonia: severe acute respiratory syndrome (SARS) and the Middle East respiratory syndrome (MERS). Since COVID-19 was first reported in December 2019 in Wuhan, China as a case of pneumonia of unknown etiology, there have been reports of more than 97 million cases and more than two million deaths worldwide as of 21st January 2021 [1]. Even though COVID-19 has affected all regions of the world, there are great variations in the prevalence of the disease and mortality rates in different countries, the reasons for which are poorly understood. For example, in China, where the first case of COVID-19 was reported and which has the largest population in the world, the prevalence has been very low compared to other countries.

In the case of India, where more than 10.5 million cases of COVID-19 have been reported till 21st January 2021, the mortality rate has been low compared to the western countries [1]. Even within India, different states have reported different patterns of disease manifestation; for example, some states like Maharashtra, Delhi, Tamil Nadu, and Andhra Pradesh have been severely affected and also reported cases at the beginning of the pandemic itself. However, other states, especially those in Northeast India, have reported a very low prevalence of COVID-19, and moreover, they started reporting cases much later in comparison to the rest of India [2]. Thus, it seems that COVID-19 manifests differently in different regions of the world. These differences may be attributed to various factors, such as variations in the mean age of the population, differences in ethnic backgrounds, and prevalence of comorbidities, which need to be further evaluated [3-4]. Although some articles on COVID-19 have been published from different parts of India [5-7], research on the clinical profile of COVID-19 patients from Northeast India, which has a different socio-cultural and demographic profile from the rest of the country, has been scarce [8-9]. In light of this, we conducted the present study to highlight the patterns related to COVID-19 in Northeast India.

## Materials and methods

This was a hospital-based prospective observational study carried out from July 2020 to October 2020 at the Department of General Medicine, North Eastern Indira Gandhi Regional Institute of Health and Medical Sciences (NEIGRIHMS), a tertiary care hospital in Shillong, Meghalaya. We included only laboratory-confirmed cases of COVID-19 admitted to our institute during the study period, and cases with incomplete data as per the proforma were excluded. Patients were grouped into the following categories as per the institute's management protocol:

1. Asymptomatic case: a laboratory-confirmed case of COVID-19 without any signs and symptoms.

2. Mild case: a laboratory-confirmed case with one or more of the following signs and symptoms but without shortness of breath and normal SpO_2_ (>94%) on room air: fever, cough, malaise, rhinorrhea, anosmia, sore throat, or lethargy.

3. Moderate case: a laboratory-confirmed case with any of the following features: respiratory rate >24/minute, SpO_2_ <95% in room air, altered sensorium, or hypotension [systolic blood pressure (SBP) <90 mmHg or diastolic blood pressure (DBP) <60 mmHg].

4. Severe case: a laboratory-confirmed case with any of the following features: respiratory failure, shock, or multi-organ dysfunction.

Patient care was provided at three levels based on the patients' severity and associated comorbidities as follows:

1. Level A COVID-19 (isolation ward): asymptomatic and mild cases.

2. Level B COVID-19 [high dependency unit (HDU) care facility]: moderate cases.

3. Level C COVID-19 (ICU care facility): severe cases.

All patients were monitored throughout their hospital stay and followed up after discharge in a COVID-19 follow-up outpatient department (OPD) or through telemedicine consultation. Data related to the patient’s demographic profile, clinical parameters, and treatment were collected on a predesigned proforma and entered into a Microsoft Excel 2019 sheet. The patient data captured were as follows: age, gender, exposure history, categories (healthcare worker or not), clinical profile, comorbidities, complications, outcomes, and duration of hospital stay. Continuous variables are expressed in the form of mean with standard deviation. A Chi-square test was done to find the significance of the difference of measured variables in different categories. A p-valve of <0.05 was considered statistically significant.

Ethical approval was obtained from the Institution Ethics Committee, North Eastern Indira Gandhi Regional Institute of Health and Medical Sciences vide letter No M12/F28/2020, and informed written consent was taken from all participants in the study.

## Results

The data of a total of 180 patients who were admitted during the study period from 1st July to 31st October 2020 were analyzed. Of the total 180 cases, 104 (57.78%) were male and 76 (42.22%) were female with a male-to-female ratio of 1.37:1. The age of the patients ranged from 18 to 80 years (mean age: 37.17 years) (Table 1).

The median (IQR) duration of symptoms was four days (two to five days) before admission to the hospital. Of all the cases, 139 (75.96%) belonged to the general population, and 44 (24.04%) were healthcare workers. A total of 101 (56.11%) cases had some risk factors for transmission of COVID-19 but 79 (43.89%) did not have any known risk factors or a history of contact with COVID-19-positive patients. Out of 101 patients with risk factors, nine (5%) had a history of recent domestic travel to or from affected states while 92 (51.11%) were exposed to a laboratory-confirmed COVID-19 case. Among these 92 patients, 51 (28.33%) cases had exposure at the workplace and 41 (22.78%) had exposure within their families.

Of the total cases, 102 (56.67%) were asymptomatic and remained so throughout the period of hospital stay. Among the 78 (43.33%) symptomatic cases, 26 (14.44%) had mild disease, 18 (10%) had moderate disease, and 34 (18.89%) had severe disease (Table 1). Although a wide index of suspicion is required to diagnose COVID-19, a constellation of presenting symptoms such as fever (n=55, 70.51%), cough (n=42, 53.85%), breathlessness (n=32, 42.02%), fatigue (n=29, 37.12%), headache (n=24, 30.77%), nasal congestion (n=19, 24.36%), nausea and/or vomiting (n=15, 19.23%), abdominal pain (n=10, 12.82%), and loss of smell (n=10; 12.82%) were found to be useful indicators for the diagnosis (Table 2). Although people were equally likely to manifest symptoms across all age groups, severe symptoms were more commonly seen in elderly patients and most of the younger population had asymptomatic presentations (Table 3).

**Table 1:** Clinical characteristics of 78 symptomatic patients from Northeast India with COVID-19 COVID-19: coronavirus disease 2019; IQR: interquartile range

Clinical characteristics	Values
Duration of symptoms, days, median (IQR)	4 (2-5)
Symptom at admission, n (%)	
Fever (>38 °C)	55 (70.51%)
Cough	42 (53.85%)
Breathlessness	32 (41.02%)
Fatigue	29 (37.12%)
Headache	24 (30.77%)
Sputum	21 (26.92%)
Nasal congestion	19 (24.36%)
Sore throat	16 (20.51%)
Nausea and vomiting	15 (19.23%)
Diarrhea	12 (15.38%)
Loss of smell	10 (12.82%)
Pain in the abdomen	10 (12.82%)
Insomnia	8 (10.26%)
Skin rash	3 (3.85%)
Outcome (n=180), n (%)	
Recovered and discharged	145 (80.56%)
Shifted to the corona care center	6 (3.33%)
Discharged to other facilities	2 (1.11%)
Expired	27 (15%)

**Table 2:** Demographics and baseline characteristics of 180 patients from Northeast India with COVID-19 COVID-19: coronavirus disease 2019

Variables	Values
Age in years	
Mean	37.17
≤20, n (%)	6 (3.33%)
21-30, n (%)	59 (32.78%)
31-40, n (%)	44 (24.44%)
41-50, n (%)	25 (13.89%)
51-60, n (%)	23 (12.78%)
≥60, n (%)	23 (12.78%)
Gender, n (%)	
Male	104 (57.78%)
Female	76 (42.22%)
Epidemiological features, n (%)	
No contact history	79 (43.89%)
Contact with laboratory-confirmed COVID-19 case	92 (51.11%)
History of domestic travel to affected areas	09 (5%)
Clinical classification, n (%)	
Asymptomatic	102 (56.67%)
Mild disease	26 (14.44%)
Moderate disease	18 (10%)
Severe disease	34 (18.89%)
Healthcare workers (n=44), n (%)	
Doctors	11 (25%)
Nursing staff	15 (34%)
Technicians	4 (9.1%)
Ward attendants	5 (11.4%)
Other supporting staff	9 (20.5%)

**Table 3:** Age distribution of 180 patients from Northeast India with COVID-19 based on clinical classifications COVID-19: coronavirus disease 2019

Age group (years)	Asymptomatic, n (% of asymptomatic patients)	Mild disease, n (% of mild patients)	Moderate disease, n (% of moderate patients)	Severe disease, n (% of severe patients)	Total, n (% of total cases)
≤20	5	0	1	0	6
21-30	45	10	2	2	59
31-40	35	6	2	1	44
41-50	11	4	4	6	25
51-60	2	4	5	12	23
>60	4	2	4	13	23
Total	102 (56.67%)	26 (14.44%)	18 (10.00%)	34 (18.89%)	180 (100%)

**Figure 1:**
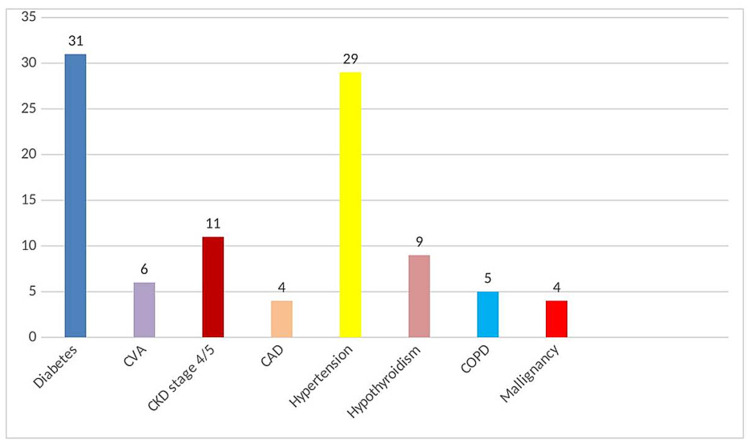
Comorbidities associated with COVID-19 patients COVID-19: coronavirus disease 2019; CVA: cerebrovascular accident; CKD: chronic kidney disease; CAD: coronary artery disease; COPD: chronic obstructive pulmonary disease

Comorbidities were present in 84 (46.67%) cases; diabetes mellitus (n=31; 36.9%) was the most common comorbidity observed followed by hypertension (n=29, 34.52%) and chronic kidney disease (n=11, 14.1%) (Figure 1). Patients with advanced age and comorbidities were found to have the severe form of the disease (Table 4). As per the Chi-square test, it was found that symptomatic disease was significantly higher among cases with comorbidities (p<0.00001).

Out of 180 cases, 145 (80.56%) patients recovered and were discharged; six (3.33%) patients were shifted to the corona care center and two (1.11%) were discharged to other facilities. A total of 27 (15%) patients expired during their hospital stay. On correlating the age of the patients with their outcomes, we found that the average age of patients who expired (55.15 ±16.34 years) was significantly higher than those who survived (37.16 ±14.22 years, p<0.0001).

**Table 4:** Categories of 180 patients from Northeast India with COVID-19 based on clinical classification and its relation to age and comorbidities COVID-19: coronavirus disease 2019; SD: standard deviation

Categories	Age in years, mean ±SD	Comorbidities, n (%)
Asymptomatic	33.01 ±11.37	25 (24.5%)
Mild symptoms	38.23 ±14.15	8 (30.76%)
Moderate symptoms	47.61 ±16.86	11 (61.11%)
Severe symptoms	57.26 ±12.67	32 (94.18%)

## Discussion

This was a hospital-based study, and it is expected that the clinical and demographic profile of the patients would be different from that of a community-based study. Our study was conducted at a tertiary care teaching institute and an apex referral center in the state of Meghalaya, India. In comparison to the rest of India, the Northeastern region was affected much later by the COVID-19, and states in Northeastern India are still among the least affected states of India [2,10]. The first case of COVID-19 in Meghalaya was diagnosed on 13th April 2020, which was much later after 30th January 2020, when the first case of COVID-19 was reported in India [11]. The present study included only those patients admitted to our facility from 1st July to 31st October 2020. The proportion of males was found to be slightly higher than females, which is similar to the findings of the other studies from India [12]. The reason for male predominance in most of the studies may be due to the fact that males tend to travel more and are more actively engaged in outdoor activities during the lockdown period as compared to females. Some studies also show that males suffered from more severe forms of COVID-19 than females and this may explain why there were more males among the hospitalized patients than females in the present study [13-14]. The mean age of affected males (37.17 years) was much lower than that of females (41.81 years), which may be attributed to the fact that young males may get exposed to the disease more often as they are more involved in outdoor activities.

The overall mean age of the affected cases was 39.85 years, which is very similar to the findings from other studies but is in stark contrast with the data from the western world. Young people are generally more engaged in work during the lockdown period, which might increase their chances of getting exposed to COVID-19, while older people prefer to stay at home [12]. Close contact with a known case of COVID-19, symptomatic or asymptomatic, is the main source of transmission [15]. In the present study, only 56.11% of patients had some risk factors for COVID-19 exposure, such as contact with a confirmed case of COVID-19, travel history outside the state in the recent past, or being a healthcare worker involved in COVID-19 patient care. Almost one-fourth of the cases (24.04%) were healthcare workers of which the most common group affected was nursing officers followed by clinicians and non-treating support staff. Similar findings were reported from Qatar where the nursing staff was found to be the most common group affected by COVID-19 [16]. Among the healthcare workers who were diagnosed with COVID-19, the majority were not directly involved in patient care in COVID-19-designated areas.

Being a hospital-based study, the true proportion of COVID-19 patients based on different categories of severity could not be assessed in a way that could be extrapolated to the general population; however, from our findings, we can still draw inferences about the effect of age and comorbidities on the severity and admission pattern. The present study shows that as the age of the patient increases, the chance to have more severe disease increases as well [17]. The young population is more likely to be asymptomatic, and similar finding has been reported from other studies [18]. Associated comorbidities are an important factor to determine the disease outcomes [19]. Our study found that patients with comorbidities, particularly diabetes and/or chronic kidney disease, had more severe disease and high mortality, which is highly significant when compared to the patient without comorbidities. Similar findings were noted in other studies and the most common associated comorbidities observed were diabetes, coronary artery disease, chronic obstructive pulmonary disease, and chronic kidney disease [19-20]. The in-hospital mortality rate in the present study is 15%, which is much higher than the mortality of overall COVID-19 patients as more patients with severe diseases and comorbidities were admitted. Being a tertiary care center, our facility also receives critical patients referred from other hospitals in the region, which could have introduced a referral bias in our findings. The present study also shows that male patients have higher mortality rates in comparison to females, which is in line with findings from many other studies [21-22].

## Conclusions

We documented the epidemiological characteristics and clinical manifestation of COVID-19 among patients from Northeast India. We also focused on comorbidities associated with COVID-19 patients, which can help clinicians in early screenings of high-risk patients and judicious utilization of healthcare resources among these patients to prevent the more severe disease. As the COVID-19 pandemic unfolds, more research and studies are the need of the hour. Further extensive studies with short- or long-term follow-up assessments with larger sample sizes from different regions of the world are required to gain deeper insights into COVID-19, which would lead to a better characterization of the course of this disease.
